# Cytokine and chemokine profiles linked to early severity of scrub typhus: multicenter validation of soluble PD-L1

**DOI:** 10.1128/jcm.01633-25

**Published:** 2026-04-27

**Authors:** Dachuan Lin, Gaoyu Wang, Liyuan Zhang, Meng Chang, Hua Pei, Xiaoyuan Hu, Ruoyan Peng, Chuanning Tang, Yijia Guo, Siqi Chen, Nan Ge, Wenjun Tian, Fahui Wang, Yongguo Du, Shannan Wu, Yueping Wang, Long Sun, Biao Wu, Kwok-Yung Yuen, Jasper Fuk-Woo Chan, Min Liao, Feifei Yin

**Affiliations:** 1 Hainan Medical University-The University of Hong Kong Joint Laboratory of Tropical Infectious Diseases and Academician Workstation of Hainan Province, Key Laboratory of Tropical Translational Medicine of Ministry of Education, School of basic medical sciences, Hainan Academy of Medical Sciences, Hainan Medical University 12455, Haikou, Hainan, China; 2 Department of Pathogen Biology, Hainan Medical University 12455, Haikou, Hainan, China; 3 Department of Infectious Diseases, the Second Affiliated Hospital of Hainan Medical University 477165 , Haikou, Hainan, China; 4 Department of Clinical Laboratory, Center for laboratory Medicine, Hainan Women and Children’s Medical Center, Hainan Medical University 12455, Haikou, Hainan, China; 5 Animal and Plant Quarantine Center Haikou Customs District, Haikou, Hainan, China; 6 Department of Infectious Diseases, the First affiliated Hospital of Hainan Medical University 607156, Haikou, Hainan, China; 7 Department of Infectious Diseases, Hainan General Hospital 26496, Haikou, Hainan, China; 8 Hainan Public Health Clinical Center, Haikou, Hainan, China; 9 State Key Laboratory of Emerging Infectious Diseases, Carol Yu Center for Infection, Department of Microbiology, School of Clinical Medicine, Li Ka Shing Faculty of Medicine, The University of Hong Kong, Pokfulam, Hong Kong, China; 10 Department of Infectious Diseases and Microbiology, The University of Hong Kong-Shenzhen Hospital 444333, Shenzhen, Guangdong, China; 11 Department of Microbiology, Queen Mary Hospital, Pokfulam, Hong Kong, China; Mayo Clinic Minnesota, Rochester, Minnesota, USA

**Keywords:** cytokines, disease severity, scrub typhus, sPD-L1

## Abstract

Early identification of patients at risk of developing severe disease remains a clinical challenge for scrub typhus. We aimed to identify serum cytokine profiles associated with disease severity and validate potential biomarkers for early risk stratification of patients with scrub typhus. We conducted a retrospective multicenter study to analyze serum cytokine/chemokine profiles and clinical data of 51 scrub typhus patients and 13 control patients at presentation to the hospital. We further performed longitudinal monitoring of the cytokine/chemokine dynamics in 11 of these patients. The findings were then validated in two different hospitals (*n* = 88). Of 44 cytokines/chemokines tested, 32 were detectable in the serum samples of the patients. Compared with control patients, patients with scrub typhus showed significant elevation of 22 and reductions of 3 cytokines/chemokines (*P* < 0.05). The serum soluble programmed death-ligand 1 (sPD-L1) levels differed significantly across severity groups (*P* = 0.04) and trended lower during recovery (*P* = 0.082). sPD-L1 demonstrated a significant trend with increasing severity and showed superior performance to conventional markers in distinguishing patients with organ dysfunction. Receiver operating characteristic analysis of sPD-L1 levels at hospital admission confirmed high discriminatory power, with AUCs of 0.997 and 0.981 in the two validation cohorts. This multicenter study identified sPD-L1 as a potential early biomarker for early risk stratification of patients with scrub typhus.

**IMPORTANCE:**

Scrub typhus, caused by *Orientia tsutsugamushi*, affects over one million people annually in the Asia-Pacific region, with an in-hospital mortality rate of more than 30% among patients with severe disease. Early identification of patients at high risk of progression to multi-organ failure remains a major clinical challenge. Timely risk stratification to identify high-risk patients is essential to prevent poor clinical outcomes. This study identifies soluble programmed death-ligand 1 as a potential early biomarker that distinguishes patients at risk of developing severe disease within the first week of hospitalization.

## INTRODUCTION

Scrub typhus is a leading cause of acute undifferentiated fever in Asia (1). Scrub typhus, caused by *Orientia tsutsugamushi* (*Ot*), is an acute febrile illness that ranges in severity from subclinical or minimally symptomatic infection (detected primarily through serological surveys in endemic areas) to self-limiting febrile illness, and in a subset of patients, progression to severe multi-organ complications and death (2–6). While the overall in-hospital mortality rate is approximately 5%, it can exceed 30% in patients with severe disease (7–9). Therefore, early risk stratification is critical to enable timely intervention, including the use of appropriate antibiotics and intensive supportive care to prevent disease progression.

Despite the clinical significance of scrub typhus, the ability to predict disease severity during the initial stages remains limited. While inflammatory cytokines/chemokines have been proposed as potential biomarkers for assessing disease severity and prognosis (10, 11), the majority of existing studies have focused on immune characteristics during the later stages of infection or post-treatment phases (12, 13). Little is known about the cytokine/chemokine dynamics during the critical early phase of disease. This study addresses this gap by analyzing immune markers of different disease severity groups at comparable stages and identifies potential biomarkers to allow early recognition of patients at risk of developing severe disease.

## MATERIALS AND METHODS

### Study population and procedures

We retrospectively reviewed medical records and serum samples of patients admitted to four major hospitals in Hainan Province between 1 January 2019 and 31 December 2024. The patients were included if they met clinical and laboratory criteria for scrub typhus. Disease severity was determined based on clinical progression with stratification based on the number of days from clinical manifestation onset to hospital admission: mild (no organ dysfunction), moderate (one organ dysfunction), or severe (≥2 organ dysfunctions) as previously outlined (14, 15). The specific criteria for organ dysfunction were as follows: (i) Respiratory: bilateral pulmonary infiltrates plus PaO₂/FiO₂ ≤ 250 mmHg, respiratory rate ≥30/min, or mechanical ventilation; (ii) Cardiovascular: systolic blood pressure <90 mmHg requiring vasopressors after fluid resuscitation, or diagnosis of myocarditis/ischemic heart disease/heart failure; (iii) Central nervous system: altered mental status, seizure, or intracranial hemorrhage/infarction; (iv) Renal: serum creatinine ≥177 µmol/L (2.0 mg/dL) or requirement for renal replacement therapy; (v) Hepatic: total bilirubin ≥42.7 µmol/L; (vi) Digestive: clinically diagnosed gastrointestinal hemorrhage. A dedicated grouping strategy was applied across cohorts: (i) Discovery cohort: retained the three-tiered classification (mild/moderate/severe) due to sufficient subgroup sample sizes (mild *n* = 38, moderate *n* = 8, and severe *n* = 5), enabling analysis of severity gradients; (ii) Validation cohorts: merged moderate and severe cases into a “high-risk group” (vs. mild “low-risk group”) for two reasons: (i) Clinical objective—early screening of patients requiring urgent intervention (any organ dysfunction warrants escalated care); (ii) Statistical feasibility—only three severe cases (≥2 organ dysfunctions) were enrolled during the 1-year validation period, precluding powered three-group comparisons. The underlying medical definition of organ dysfunction remained consistent across all cohorts. A dedicated grouping strategy was applied across cohorts to balance clinical relevance and statistical feasibility: (i) Discovery cohort: Retained the three-tiered severity classification (mild/moderate/severe) due to sufficient sample sizes in each subgroup (mild *n* = 38, moderate *n* = 8, and severe *n* = 5), enabling analysis of severity gradients; (ii) Validation cohorts: Merged moderate and severe cases into a “high-risk group” (vs. mild “low-risk group”) for two key reasons: (i) Clinical objective: The study’s core goal is early screening of high-risk patients (requiring urgent intervention) rather than grading severity among high-risk individuals; (ii) Sample size constraints: with only one severe case from the First Affiliated Hospital of Hainan Medical University (FAHHMU) cohort and two from the Second Affiliated Hospital of Hainan Medical University (SAHHMU) cohort enrolled during the 1-year validation period, the study lacked sufficient sample size for statistically powered subgroup analyses. The original severity definition (based on organ dysfunction count) remained consistent across all cohorts. Serum samples for cytokine/chemokine analysis were collected at hospital admission, before clinical deterioration. The discovery cohort was recruited from the People’s Hospital of Haikou City in the provincial capital and the People’s Hospital of Qiongzhong and Miao Autonomous County in central Hainan. The validation cohort was prospectively enrolled from two teaching hospitals—the FAHHMU and the SAHHMU, both located in Haikou.

### Multiplex assay

Upon admission, whole blood and serum samples were promptly collected and transported to the laboratory within 48 h via a cold chain, where they were stored at −80°C. Genomic DNA was extracted from whole blood samples using the QIAamp DNA Mini Kit (QIAGEN, Hilden, Germany). Nested PCR was performed with primers designed to amplify a 483 bp segment of the 56 kDa type-specific antigen (TSA) gene, following established protocols detailed in our previous research and by other scholars (15, 16). Positive PCR products were purified and subsequently subjected to Sanger sequencing. The quantity of *Ot* DNA was determined via a standard curve derived from serial 10-fold dilutions of a plasmid harboring the 56 kDa TSA gene fragment, as previously described (15). To discern the variations in serum cytokines between the acute and convalescent stages, serum specimens were collected from 11 confirmed cases prior to therapy and once more at the 7-day post-treatment mark.

### Cytokine/chemokine measurement

To elucidate the serum cytokine/chemokine profile in individuals suffering from *Ot* infection during both the acute and convalescent stages, cytokine/chemokine levels were measured using a customized Magnetic Luminex Performance Assay kit (R&D Systems, Human XL Cytokine Discovery Panel, FCSTM18, R&D Systems, Abingdon, UK), according to the manufacturer’s instructions. The data were collected on a calibrated and validated Luminex MAGPIX apparatus (R&D Systems, Abingdon, UK). Concentrations were determined using standard curves, employing a five-parameter logistic curve fit. The cytokines/chemokines investigated include: CD40 Ligand, Epidermal Growth Factor (EGF), Eotaxin, Fibroblast Growth Factor Basic, Fms-Related Tyrosine Kinase 3 Ligand (Flt3 Ligand), Granulocyte Colony-Stimulating Factor (G-CSF), Granulocyte-Macrophage Colony-Stimulating Factor (GM-CSF), Granzyme B, Growth-Regulated Oncogene-alpha (GRO-α), GRO-β, Interferon-alpha (IFN-α), IFN-β, IFN-γ, Interleukin-1 alpha (IL-1α), IL-1β, IL-1 receptor antagonist (IL-1ra), IL-2, IL-3, IL-4, IL-5, IL-6, IL-7, IL-8, IL-10, IL-12 p70, IL-13, IL-15, IL-17A, IL-17E, IL-33, Interferon Gamma-Induced Protein 10 (IP-10), Monocyte Chemoattractant Protein-1 (MCP-1), Macrophage Inflammatory Protein-1 alpha (MIP-1α), MIP-1β, MIP-3α, MIP-3β, Platelet-Derived Growth Factor-AA (PDGF-AA), PDGF-AB/BB, soluble programmed death-ligand 1 (sPD-L1), Regulated on Activation, Normal T-cell Expressed, and Secreted (RANTES), Transforming Growth Factor-alpha (TGF-α), Tumor Necrosis Factor-alpha (TNF-α), TNF-related Apoptosis-Inducing Ligand (TRAIL), and Vascular Endothelial Growth Factor (VEGF).

The concentration of sPD-L1 in human serum was measured using the Human Programmed Death-Ligand 1 ELISA Kit (Solarbio, SEKH-0402) according to the manufacturer’s protocol.

Statistical analyses were carried out using GraphPad PRISM Version 9.0 (GraphPad Software, CA, USA). Data normality was assessed using the Kolmogorov-Smirnov test prior to analysis. For comparisons between scrub typhus patients and controls (intergroup), unpaired *t*-tests (parametric) or Wilcoxon rank-sum tests (non-parametric) were used. For comparisons between acute and recovery phases in the same patients (intragroup), paired *t*-tests (parametric) or Wilcoxon signed-rank tests (non-parametric) were applied.

For normally distributed data, comparisons were conducted using the *t*-test for the control group with the scrub typhus patient group and paired samples *t*-test for comparisons between infection recovery groups and one-way analysis of variance (ANOVA) for comparisons among multiple groups. For non-normally distributed data, comparisons were conducted using the Wilcoxon test and the Kruskal-Wallis test for comparisons among multiple groups. For receiver operating characteristic (ROC) analyses, the null hypothesis was that the area under the curve (AUC) equals 0.5 (i.e., no discriminatory ability between severe and non-severe cases), and the alternative hypothesis was that AUC ≠ 0.5. For validation cohort analyses, “severe” was defined as the high-risk group (moderate + severe, i.e., any organ dysfunction) vs. low-risk (mild), consistent with the binary stratification strategy described above.

## RESULTS

### Patient characteristics

Among 51 scrub typhus patients, clinical manifestations ranged from mild fatigue to severe multi-organ dysfunction (Table 1). The cohort included 13 controls, 38 mild cases, 8 moderate cases, and 5 severe cases. Longitudinal monitoring tracked cytokine/chemokine dynamics in 11 patients, including 2 with moderate disease. Organ dysfunction was a defining feature of the 13 patients with moderate and severe disease (consistent with our severity criteria). Specifically, liver dysfunction was observed in five patients, kidney dysfunction in four, central nervous system involvement in four, respiratory dysfunction in three, and circulatory dysfunction in three (Table S1). Age and gender distributions were similar across organ failure groups (Tables S1 and S2). Among all 51 patients, fever (96%) and headache (51%) were the most dominant clinical manifestations, while eschars appeared in only 37% of cases (40% in the severe group), limiting their predictive value for severity (Table 1). Other commonly reported clinical manifestations included myalgia, vomiting, lymphadenopathy, and skin rash, which were similarly distributed across disease severity categories.

**TABLE 1:** Clinical manifestations of patients with scrub typhus and control subjects

		Control	Mild	Moderate	Severe
Demographics and clinical manifestations	Age-year	53.8 ± 13.2	51.6 ± 14.8	56.0 ± 13.7	49.0 ± 25.1
	Male sex-No. (%)	6 (46)	24 (63)	6 (75)	2 (40)
	Renal failure No. (%)	0	0	2 (25)	2 (40)
	Hepatic dysfunction No. (%)	0	0	3 (38)	2 (40)
	Central nervous system-affection No. (%)	0	0	0	4 (80)
	Respiratory dysfunction No. (%)	0	0	2 (25)	1 (20)
	Circulatory dysfunction No. (%)	0	0	1 (13)	2 (40)
	Fever No. (%)	0	37 (97)	7 (88)	5 (100)
	Eschar No. (%)	0	14 (37)	3 (38)	2 (40)
	Headache No. (%)	0	22 (58)	4 (50)	0
	Generalized myalgia No. (%)	0	8 (21)	3 (38)	0
	Nausea, emesis No. (%)	0	3 (8)	2 (25)	0
	Lymphadenopathy No. (%)	0	1 (29)	1 (13)	0
	Skin rash No. (%)	0	2 (5)	1 (13)	0
Underlying diseases	Previously healthy No. (%)	13 (100)	24 (63)	1 (13)	4 (80)
	Diabetes mellitus No. (%)	0	5 (13)	1 (13)	0
	Malignancy No. (%)	0	0	1 (13)	0
	Chronic liver disease No. (%)	0	1 (3)	3 (38)	0
	Chronic kidney disease No. (%)	0	1 (3)	0	0
	Chronic heart disease No. (%)	0	3 (9)	0	1 (20)
	Hypertriglyceridemia No. (%)	0	2 (6)	0	0
	Hypertension No. (%)	0	3 (9)	1 (13)	0
	Cerebral infarction No. (%)	3	3 (9)	1 (13)	0

### Comprehensive cytokine, chemokine, and growth factor profiling reveals immune dysregulation in patients with scrub typhus

**Fig 1:**
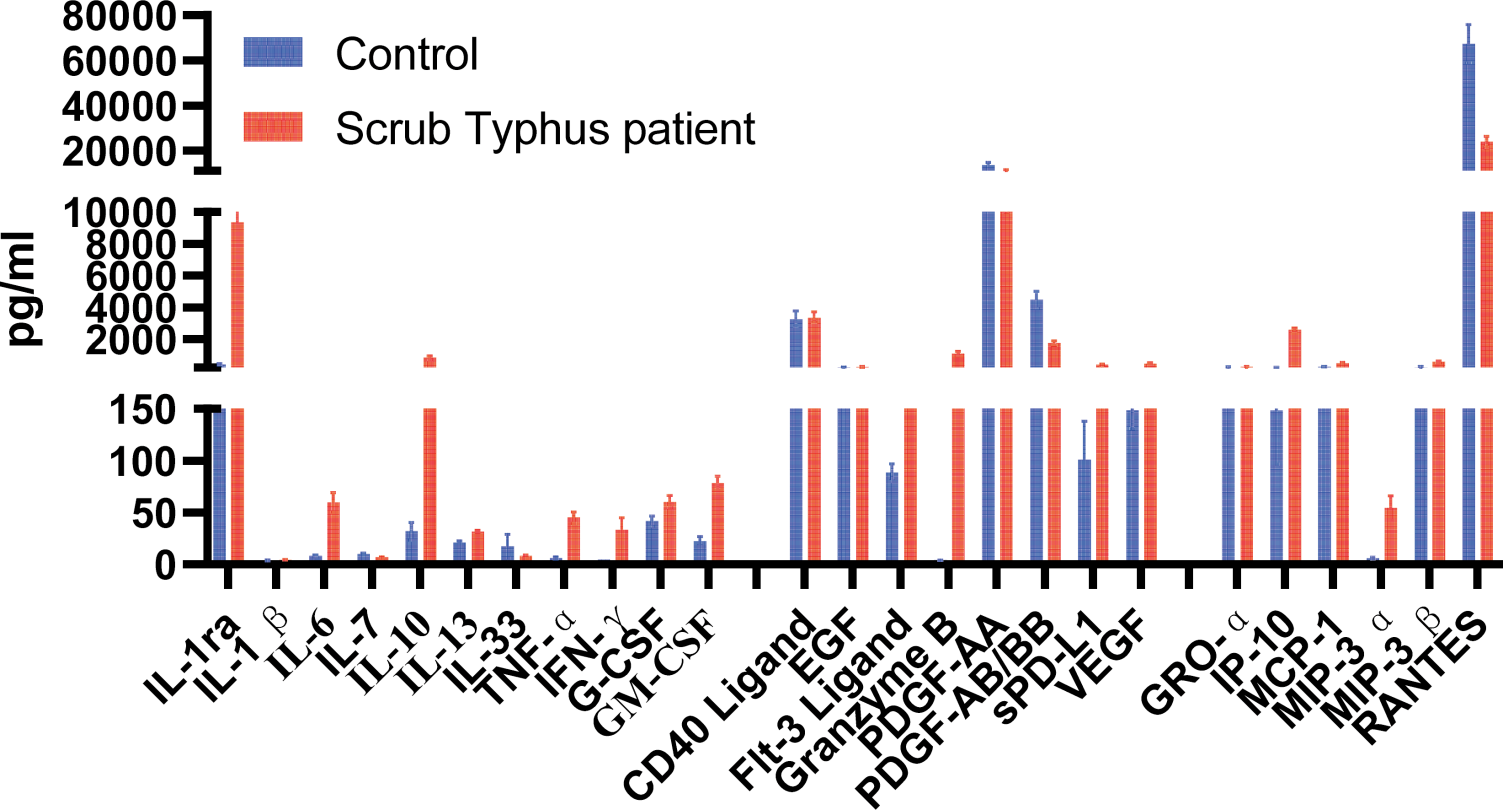
Serum cytokine profiles in scrub typhus and control patients. Data normality was assessed via Kolmogorov-Smirnov tests, with intergroup comparisons performed by unpaired *t*-tests (parametric) or Mann-Whitney *U* tests (non-parametric). Statistically significant differences (*P* < 0.05) were observed for multiple cytokines, including: Significantly elevated levels in patients: IL-6 (*P* = 0.0012), TNF-α (*P* < 0.001), IFN-γ (*P* = 0.008), IL-10 (*P* < 0.001), GM-CSF (*P* < 0.001), Granzyme B (*P* < 0.001), PD-L1 (*P* < 0.001), and VEGF (*P* = 0.002). Non-normal cytokines (all *P* < 0.05): IL-7, IL-13, IL-1ra, IL-1β, IL-33, G-CSF, CD40 Ligand, Flt3 ligand, and PDGF-AB/BB. Normal-distributed cytokines: EGF (*P* = 0.04) and PDGF-AA (*P* = 0.007).

To assess the inflammatory response, serum cytokine/chemokine levels were measured in scrub typhus patients and control participants. While 22 cytokines/chemokines were significantly elevated in scrub typhus patients compared to controls, three cytokines, namely—PDGF-AB/BB, RANTES, and IL-7—showed significantly reduced levels. Some of these cytokines and chemokines play a role in the immune clearance of *Ot*:: Elevations in IL-1ra, IL-1β, IL-6, IL-7, IL-10, IL-13, IL-33, TNF-α, IFN-γ, G-CSF, and GM-CSF were notably pronounced in patients afflicted with scrub typhus (*P* < 0.0001–0.0137), thereby highlighting their indispensable function in the inflammatory cascade. Growth Factors: Notable fluctuations were observed in CD40 Ligand, EGF, Flt3 ligand, Granzyme B, PDGF-AA, PDGF-AB/BB, sPD-L1, and VEGF (*P* < 0.05). CC and CXC chemokines: Significant disparities were discerned in GRO-α, IP-10, MCP-1, MIP-3α, MIP-3β, and RANTES (*P* < 0.05). In conclusion, scrub typhus is marked by a profound disturbance in cytokines/chemokines and growth factors, collectively shaping the immunopathological framework of the condition and mediating host immune clearance of the pathogen (Fig. 1). Notably, several cytokines demonstrated comparable serum concentrations between scrub typhus patients and control cohort (Fig. S1). Subsequent investigations have revealed that, although the concentrations of the cytokines TRAIL, IL-8, and MIP-1β do not exhibit significant disparities between scrub typhus patients and the control cohort, these cytokines manifest marked fluctuations in their concentrations during the acute and convalescent stages, as depicted in Fig. S2. As illustrated in Fig. S3, no substantial alterations were discerned in the remaining four cytokines.

### *Ot* bacterial load and inflammatory markers

**Fig 2:**
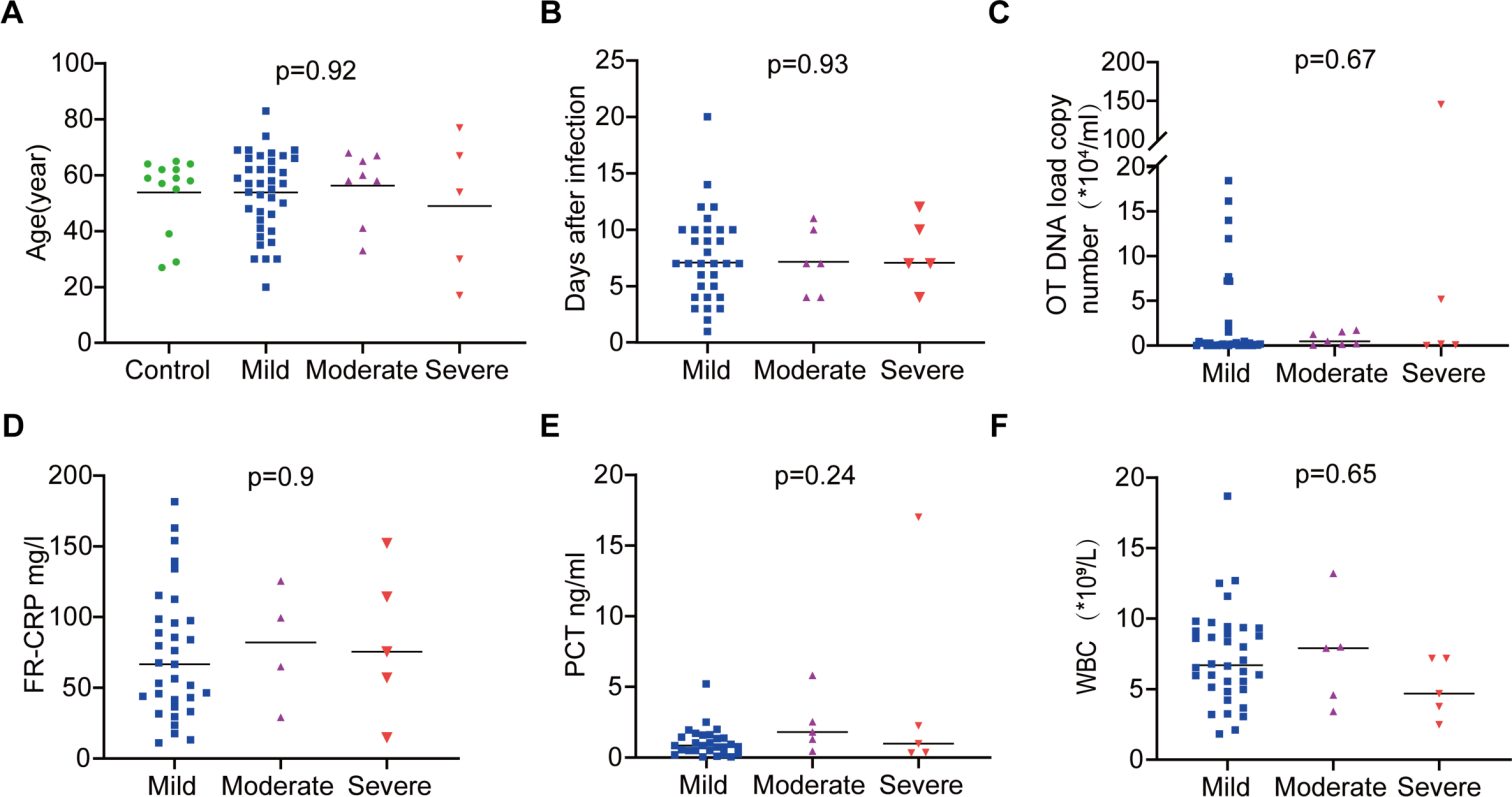
Traditional inflammatory markers and clinical parameters fail to stratify scrub typhus severity. (**A**) Age: Shapiro-Wilk normality tests—mild (*P* = 0.002), moderate (*P* = 0.32), and severe (*P* = 0.08); Kruskal-Wallis test: *P* = 0.92. (**B**) Days from onset of clinical manifestations to hospital admission: Normality—mild (*P* = 0.099), moderate (*P* = 0.33), and severe (*P* = 0.81); one-way ANOVA: *P* = 0.93. (**C**) *Ot* load: Non-normal in mild (*P* < 0.001) and severe (*P* < 0.001); Kruskal-Wallis: *P* = 0.67. (**D**) Fasting CRP: Normality—mild (*P* = 0.06), moderate (*P* = 0.93), and severe (*P* = 0.98); one-way ANOVA: *P* = 0.90. (**E**) Procalcitonin (PCT): Non-normal in severe (*P* = 0.007); Kruskal-Wallis: *P* = 0.24. (**F**) White blood cell count: Non-normal only in mild group (*P* = 0.04); Kruskal-Wallis: *P* = 0.65. Data are presented as median.

As shown in Fig. 2A, the median age was similar across groups: control (54 years [IQR: 45–63]), mild (52 years [IQR: 41–62]), moderate (56 years [IQR: 48–65]), and severe (49 years [IQR: 38–60]; *P* = 0.92, Kruskal-Wallis test). The duration from clinical manifestations onset to diagnosis was also comparable: mild (median 5 days [IQR: 4–7]), moderate (median 5 days [IQR: 4–6]), and severe (median 5 days [IQR: 4–7]; *P* = 0.93, one-way ANOVA), as depicted in Fig. 2B. Meanwhile, the *Ot* bacterial load showed high variability but no significant differences: mild (median 25,800 copies/mL [IQR: 18,500–32,100]), moderate (median 6,900 copies/mL [IQR: 4,800–9,300]), and severe (median 85,400 copies/mL [IQR: 28,600–412,000]; *P* = 0.67, Kruskal-Wallis test) as shown in Fig. 2C. Additionally, fibrinogen-reactive CRP (FR-CRP) levels were: mild (median 72.5 mg/L [IQR: 65.3–81.2]), moderate (median 78.4 mg/L [IQR: 68.9–92.1]), and severe (median 81.6 mg/L [IQR: 70.2–95.8]; *P* = 0.90, one-way ANOVA; Fig. 2D), with no significant differences between groups (*P* = 0.90, one-way ANOVA). Similarly, procalcitonin (PCT) levels displayed substantial variation, particularly in severe cases: mild (median 0.98 ng/mL [IQR: 0.75–1.35]), moderate (median 1.85 ng/mL [IQR: 1.20–3.10]), and severe (median 4.25 ng/mL [IQR: 2.15–12.80]; *P* = 0.24, Kruskal-Wallis test; Fig. 2E), with no significant differences between groups (*P* = 0.24, Kruskal-Wallis test). Finally, white blood cell counts (WBCs) were: mild (median 7.1 × 10⁹/L [IQR: 6.2–8.3]), moderate (median 7.3 × 10⁹/L [IQR: 5.8–8.9]), and severe (median 5.1 × 10⁹/L [IQR: 4.2–6.3]; *P* = 0.65, Kruskal-Wallis test; Fig. 2F), with no significant differences between the groups (*P* = 0.65, Kruskal-Wallis test). However, although *Ot* bacterial load did not differ significantly across severity groups, a trend toward higher *Ot* bacterial load correlated with elevated inflammatory markers (e.g., PCT: *P* = 0.03; FR-CRP: *P* = 0.12; Fig. S4).

### sPD-L1 as a potential biomarker for high-risk screening in scrub typhus

**Fig 3:**
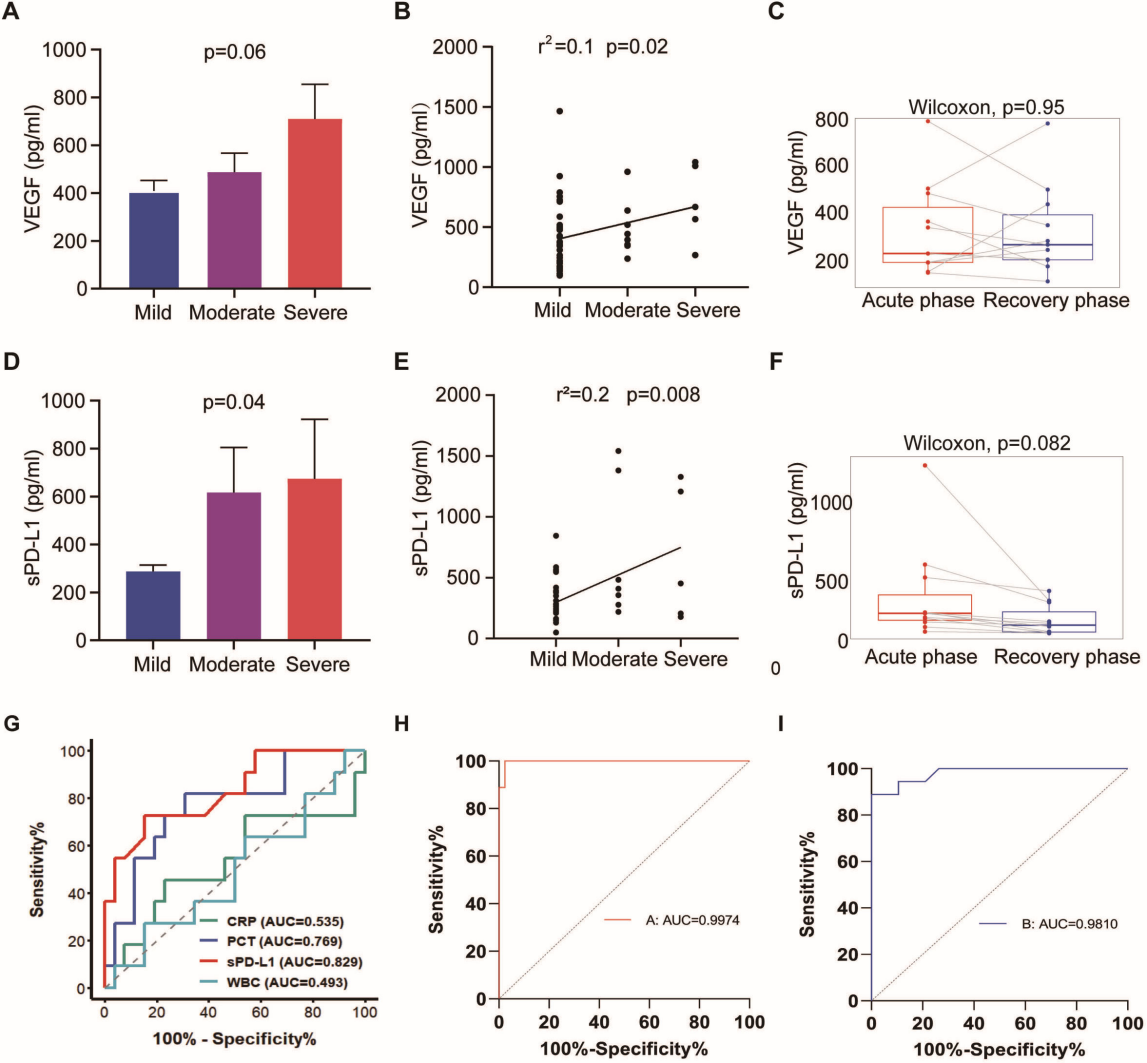
Serum VEGF and sPD-L1 levels are associated with disease severity in scrub typhus. (**A**) VEGF: Non-normal in mild (*P* < 0.001) and severe (*P* = 0.56); Kruskal-Wallis *P* = 0.057. (**B**) VEGF vs. severity: Weak positive correlation (*r*² = 0.10, *P* = 0.02). (**C**) VEGF: No difference between acute and recovery phases (*P* = 0.95, Wilcoxon). (**D**) sPD-L1: Non-normal in mild (*P* < 0.001) and moderate (*P* = 0.004); Kruskal-Wallis *P* = 0.04. Note: sPD-L1 robustly distinguishes low-risk (mild) from high-risk (moderate + severe) cases but does not differentiate moderate vs. severe disease, consistent with the study’s clinical focus. Note: sPD-L1 robustly distinguishes low-risk (mild) from high-risk (moderate + severe) cases but does not differentiate moderate vs. severe disease, consistent with the study’s clinical focus on early high-risk screening. (**E**) sPD-L1 vs. severity: Moderate correlation (*r*² = 0.20, *P* = 0.08). (**F**) sPD-L1: Significantly higher in acute vs. recovery phase (*P* = 0.0082, Wilcoxon). (**G**) ROC analysis (*n* = 51): AUCs—CRP 0.535, PCT 0.769, sPD-L1 0.829, and WBC 0.493. (**H and I**) sPD-L1 diagnostic performance: FAHHMU cohort AUC = 0.997 (standard error [SE] = 0.004, *P* < 0.001); SAHHMU cohort AUC = 0.981 (SE = 0.017, *P* < 0.001).

Among the eight growth factors analyzed, serum sPD-L1 level on hospital admission showed a significant increasing trend with disease severity and phase-specific changes (*P* = 0.04, Fig. 3D and E). Additionally, sPD-L1 exhibited phase-specific dynamics, declining significantly from the acute to the recovery phase (*P* = 0.0082, Fig. 3F). Among the eight growth factors analyzed, serum sPD-L1 level on hospital admission demonstrated a significant increasing trend with disease severity (*P* = 0.04, Fig. 3D and F). Importantly, sPD-L1 reliably distinguishes low-risk (mild, no organ dysfunction) from high-risk (moderate + severe, any organ dysfunction) scrub typhus patients, supporting its utility for early clinical risk stratification. However, sPD-L1 does not reliably differentiate moderate from severe disease among high-risk patients, which was not the primary objective of this study. Compared to traditional infection markers such as CRP, PCT, and WBC, sPD-L1 exhibited the highest accuracy in distinguishing disease severity, with an AUC of 0.829 (Fig. 3G).

In our initial exploratory analysis, we identified sPD-L1 as a potential biomarker associated with scrub typhus infection and disease severity. To further validate this finding and assess its clinical utility, we enrolled two independent validation cohorts from our two affiliated teaching hospitals FAHHMU (*n* = 51) and SAHHMU (*n* = 37). To ensure consistency with the Methods-defined grouping strategy (“low-risk” [mild] vs. “high-risk” [moderate + severe]) for two primary reasons: (i) Clinical Priority: The key application of an early biomarker is to distinguish low-risk patients from those with any organ dysfunction (moderate or severe) who are at higher risk of deterioration and require intensified care. (ii) Statistical Power: The number of prospectively enrolled “severe” patients (≥2 organ failures) in the validation cohorts was very low (FAHHMU: *n* = 1 and SAHHMU: *n* = 2), precluding a reliable three-group comparison. This binary approach enhances the clinical relevance and statistical robustness of the validation. In the FAHHMU cohort, 9 of 51 (17.6%) patients were classified as severe; in the SAHHMU cohort, 18 of 37 (48.6%) patients met the criteria for severe disease. ROC analyses were performed to evaluate the ability of sPD-L1 to distinguish severe from non-severe cases. In the FAHHMU cohort, sPD-L1 demonstrated excellent discriminatory power with an AUC of 0.997 (95% CI: 0.990–1.000, standard error [SE] = 0.004373, *P* < 0.001). Similarly strong results were observed in the SAHHMU cohort, where sPD-L1 showed an AUC of 0.981 (95% CI: 0.950–1.000, SE = 0.01726, *P* < 0.001; Fig. 3H and I).

Among the 6 CC/CXC chemokines and 11 additional cytokines analyzed, none showed significant differences across disease severity groups (Fig. S5A through C), further highlighting sPD-L1 as the only robust severity-stratifying biomarker.

### Dynamic cytokine/chemokine profiles

Analysis of 25 serum cytokines/chemokines revealed that 24 cytokines/chemokines showed no significant differences across mild, moderate, and severe scrub typhus cohorts (*P >* 0.05, Kruskal-Wallis test; Fig. 3 and Fig. S5A through C), indicating their inability to stratify disease severity. To support the validity of sPD-L1 as a disease activity-related marker, we analyzed acute-to-recovery phase changes in 11 patients: sPD-L1 levels declined significantly with clinical recovery (*P* = 0.0082, Wilcoxon signed-rank test; Fig. 3F), while most other cytokines/chemokines also showed phase-specific fluctuations (Fig. S6), confirming sPD-L1’s association with active infection.

## DISCUSSION

Current severity assessment tools for scrub typhus remain inadequate. Conventional clinical metrics—including organ dysfunction scores and inflammatory markers such as PCT, WBC, and FR-CRP—fail to reliably stratify disease severity (all *P* > 0.05), despite non-specific correlations with *Ot* load  (17). Although PCT shows modest discriminatory capacity (AUC = 0.769; Fig. 3G), its poor specificity—as evident from published data showing markedly higher levels in bacteremia vs. scrub typhus (median: 8.8 ng/mL vs. 0.6 ng/mL) (18) and lack of distinction from other rickettsial diseases  (19)—limits clinical utility. Inconsistent definitions of “severe disease” across studies further undermine biomarker performance  (20, 21). Clinical signs (e.g., eschar and duration of fever) and even *Ot* bacterial load (*P* = 0.67) also lack predictive value in our early-stage cohort, contradicting prior reports  (22, 23) but aligning with evidence that higher bacterial loads may paradoxically be associated with milder outcomes in animal models  (24) and our earlier study  (15). These findings collectively indicate that scrub typhus severity is governed more by host immune dysregulation than pathogen burden, underscoring the urgent need for novel, host-response-based stratification tools.

Cytokine storm—a dysregulated, hyperinflammatory host response. In viral diseases such as SARS-CoV-2 and Ebola, severity and mortality correlate strongly with the magnitude of cytokine dysregulation rather than viral load (25, 26). In scrub typhus, although the cytokine storm remains under-characterized. This motivated our unbiased cytokine profiling strategy. Among 44 analytes, VEGF and sPD-L1 emerged as the most robustly elevated and severity-correlated mediators. While VEGF has long been recognized for its role in angiogenesis, emerging evidence underscores its potent immunosuppressive functions—particularly through the induction of PD-L1 expression (27, 28). *In vitro*, stimulation of human umbilical vein endothelial cells with VEGF and IL-1β leads to increased secretion of sPD-L1, a key mediator of systemic T-cell exhaustion and immune evasion (29).

To our knowledge, this is the first observational study to report elevated serum sPD-L1 in patients with acute scrub typhus, a well-defined rickettsial infection caused by *Ot*. sPD-L1 levels correlate with disease severity gradient (mild < moderate < severe; Fig. 3D) and reliably distinguish low-risk (mild) from high-risk (moderate + severe) patients, offering a critical tool for early screening of life-threatening progression—consistent with the study’s clinical objective of identifying high-risk individuals requiring urgent intervention. Importantly, sPD-L1 concentrations declined rapidly following antimicrobial treatment and normalized alongside clinical recovery, suggesting a dynamic association with disease activity. These findings extend prior observations in heterogeneous sepsis populations, where sPD-1/sPD-L1 have been associated with mortality in intensive care unit settings (30), by demonstrating a consistent pattern in a single-pathogen, non-hospitalized, early-stage infection—thereby reducing confounding from polymicrobial etiology or advanced organ failure.

Our study identifies VEGF and, notably, sPD-L1 as innovative host-derived biomarkers that enable early and accurate stratification of scrub typhus severity—addressing a critical gap in current diagnostic approaches, which rely on nonspecific clinical or inflammatory markers. Unlike conventional tools, sPD-L1 differentially stratifies patients with and without organ dysfunction and offers potential for rapid risk identification at the time of hospital admission. While it does not distinguish between moderate and severe disease, its ability to identify any organ dysfunction is clinically actionable for triage decisions. This work pioneers the application of cancer-associated immune checkpoint biology to bacterial infection, revealing a new class of analytes for next-generation diagnostics. By leveraging host immune signatures rather than pathogen load, our approach promises improved specificity, earlier intervention, and a foundation for multiplexed, mechanism-informed assays—ushering in a paradigm shift toward precision prognostics in scrub typhus and potentially other infections driven by immune dysregulation.

The sPD-L1 measurement in this study employed a commercially available ELISA kit (Solarbio SEKH-0402), which requires standard laboratory equipment (microplate reader, washer) and can be completed within 4–5 h. The reagent cost per test is approximately USD 3–5, comparable to routine cytokine/chemokine assays and lower than multiplex platforms. While current automated platforms for CRP or PCT offer faster throughput, sPD-L1 ELISA is technically feasible in most secondary hospital laboratories in endemic regions. Future development of rapid lateral-flow or chemiluminescent assays could further facilitate point-of-care deployment.

This study has several limitations. First, the validation was conducted in a single high-endemicity region of southern China. Future studies in geographically diverse populations are needed to confirm the generalizability of sPD-L1. Second, sPD-L1 did not reliably distinguish moderate from severe cases among high-risk patients. This limitation likely reflects: (i) the relatively small number of severe patients (≥2 organ dysfunctions) available for subgroup analysis in the validation cohorts; and (ii) overlapping inflammatory responses between moderate and severe stages, as both reflect significant immune dysregulation. Importantly, this was not the study’s primary objective—our focus was on early screening for high-risk status (any organ dysfunction) rather than grading severity among already high-risk patients. Future multicenter studies with larger severe-case cohorts are needed to address this question. Third, the lack of a non-scrub typhus febrile control group limits the assessment of sPD-L1’s specificity for scrub typhus progression vs. severe infection in general. Future studies incorporating both diverse geographic cohorts and patients with other febrile illnesses will help to clarify the broader utility and specificity of sPD-L1 as a biomarker of severity for scrub typhus.

## Data Availability

The data sets generated during and/or analyzed during the current study are available from the corresponding author upon reasonable request.

## SUPPLEMENTAL MATERIAL

Figure S1 jcm.01633-25-s0001.tif Non-significant cytokine comparisons.

Figure S2 jcm.01633-25-s0002.tif Decreased TRAIL, IL-8, and MIP-1β levels during recovery from scrub typhus.

Figure S3 jcm.01633-25-s0003.tif No significant changes in TGF-α, Eotaxin, GRO-β, or IL-2 between acute and recovery phases.

Figure S4 jcm.01633-25-s0004.tif Association between Ot load and inflammatory markers in scrub typhus.

Figure S5 jcm.01633-25-s0005.pdf Cytokines lack severity discrimination.

Figure S6 jcm.01633-25-s0006.pdf Cytokine phase dynamics.

Tables S1 and S2 jcm.01633-25-s0007.docx Table S1: Clinical characteristics of patients with organ dysfunction. Table S2: Hepatic and renal function indices across disease severity groups.

Supplemental material legends jcm.01633-25-s0008.docx Legends for Figures S1 to S6, and Tables S1 and S2.

ASM does not own the copyrights to Supplemental Material that may be linked to, or accessed through, an article. The authors have granted ASM a non-exclusive, world-wide license to publish the Supplemental Material files. Please contact the corresponding author directly for reuse.
