# Supplementary figures and images for: Cytokine and chemokine profiles linked to early severity of scrub typhus: multicenter validation of soluble PD-L1
Source: J Clin Microbiol. 2026 Apr 27;64(6):e01633-25. doi: 10.1128/jcm.01633-25 (PMC13251376; doi:10.1128/jcm.01633-25)

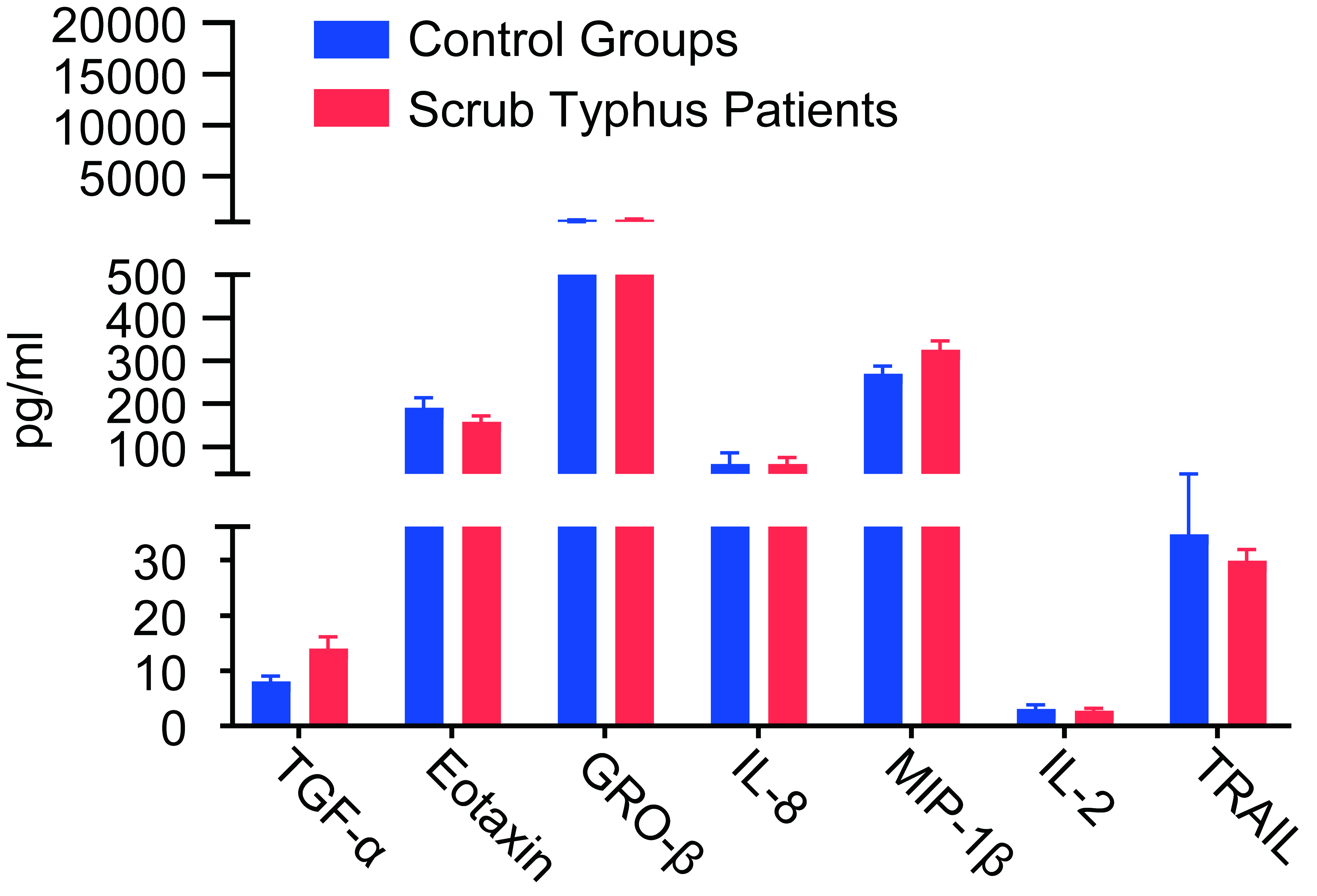

Supplement: Figure S1 — Non-significant cytokine comparisons. [file jcm.01633-25-s0001.tif]

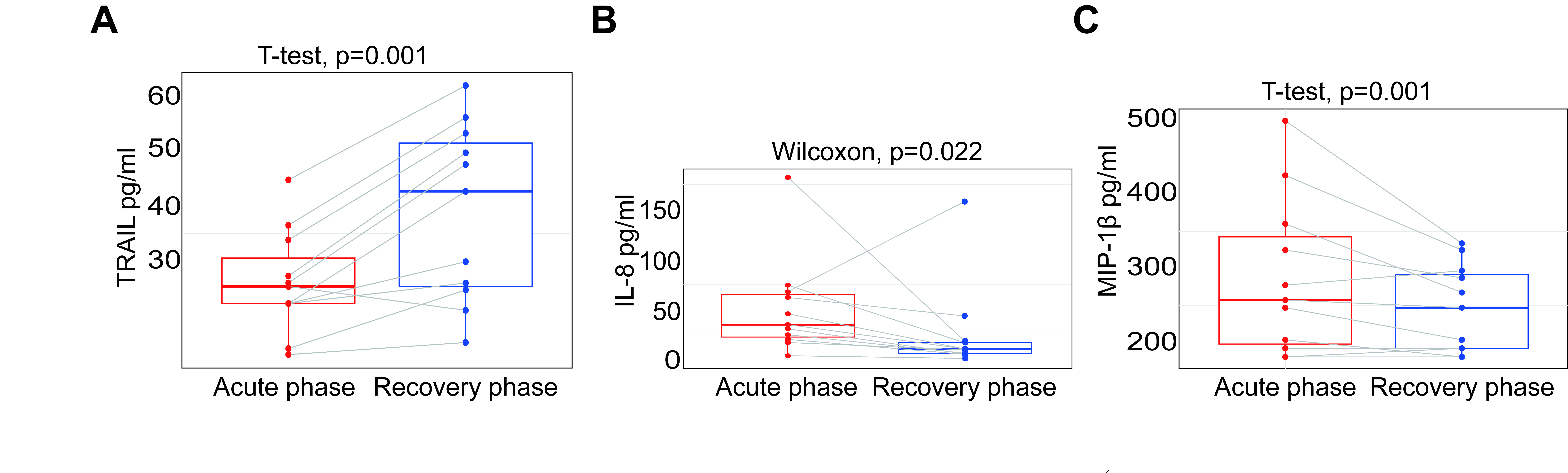

Supplement: Figure S2 — Decreased TRAIL, IL-8, and MIP-1β levels during recovery from scrub typhus. [file jcm.01633-25-s0002.tif]

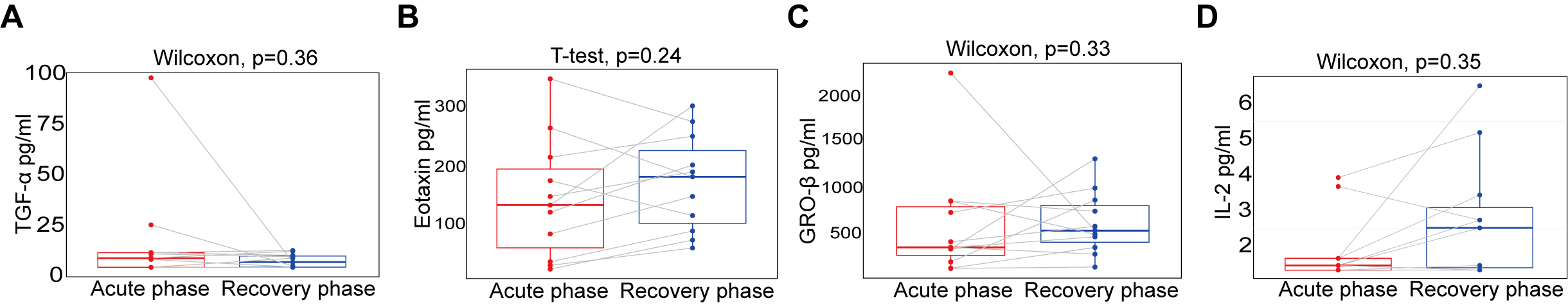

Supplement: Figure S3 — No significant changes in TGF-α, Eotaxin, GRO-β, or IL-2 between acute and recovery phases. [file jcm.01633-25-s0003.tif]

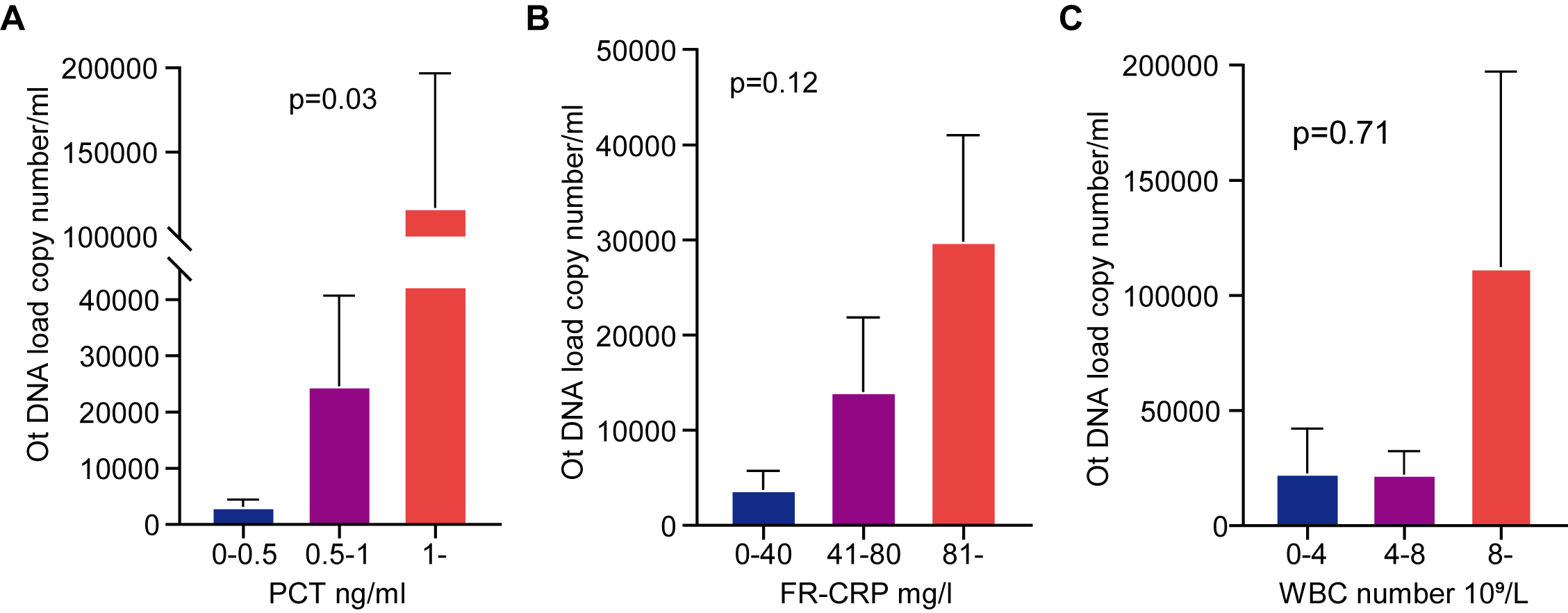

Supplement: Figure S4 — Association between Ot load and inflammatory markers in scrub typhus. [file jcm.01633-25-s0004.tif]

A-A

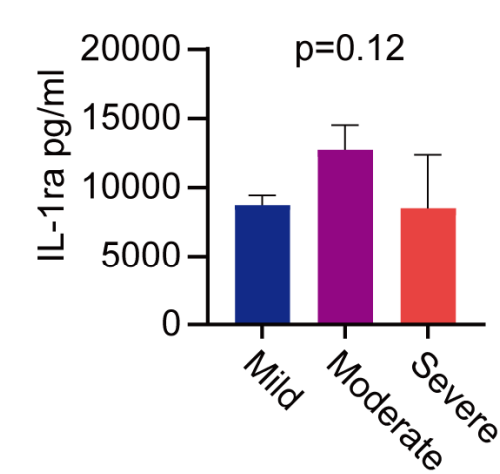

A-B

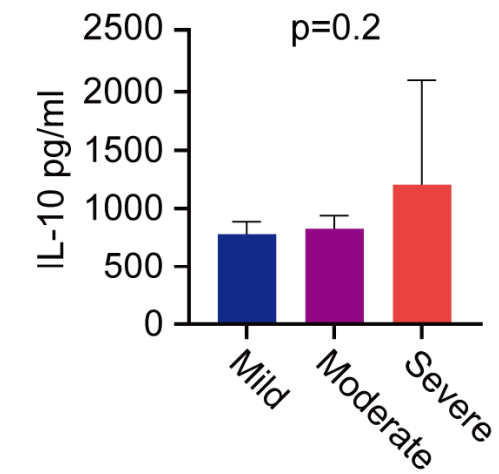

A-C

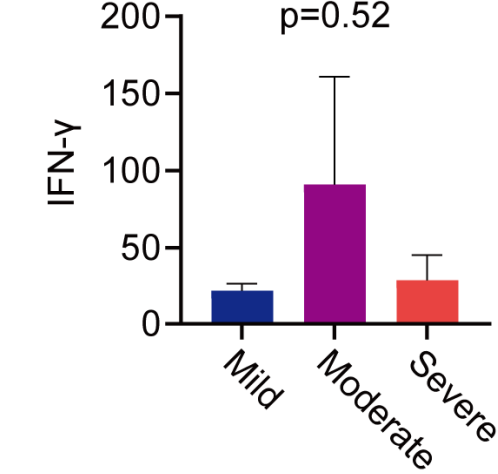

A-D

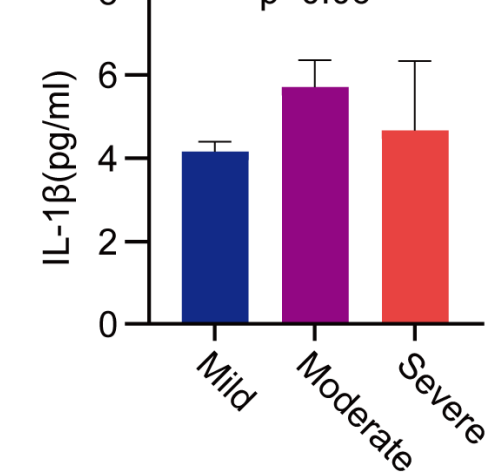

A-E

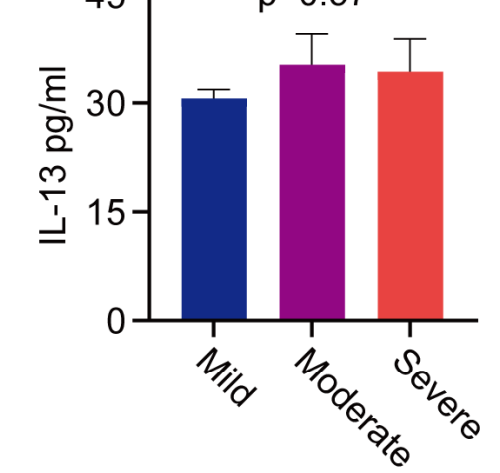

A-F

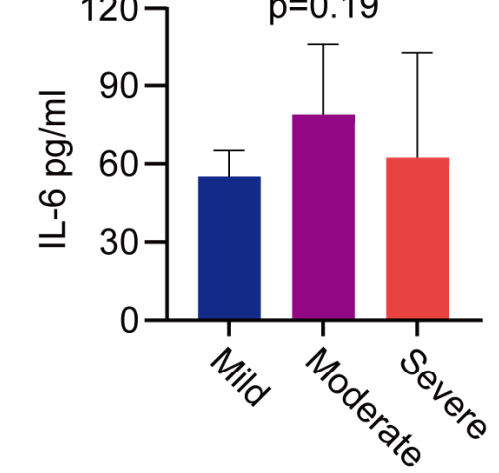

A-G

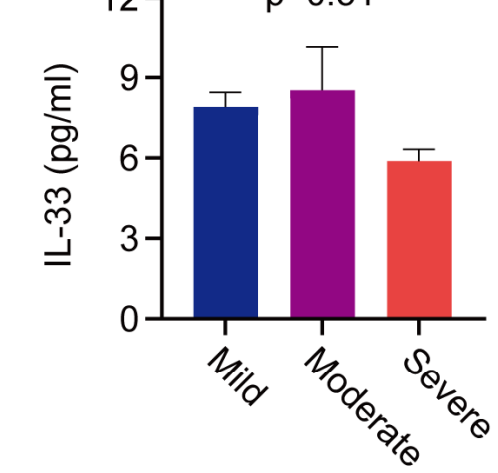

A-H

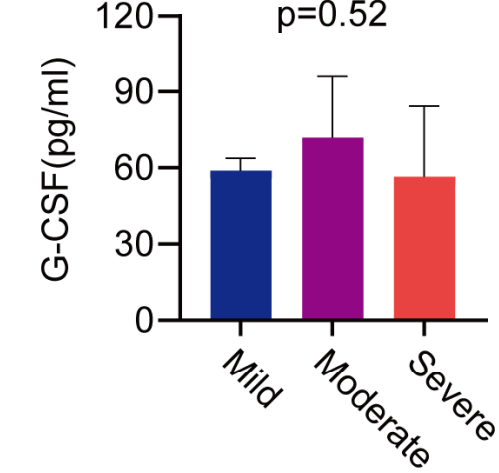

A-I

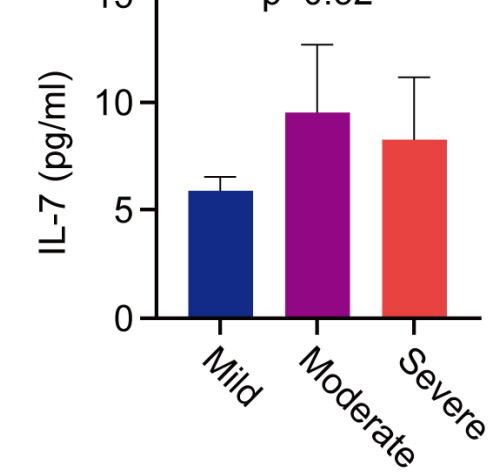

A-J

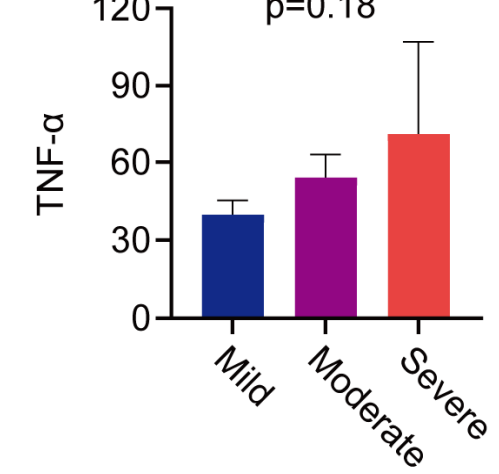

A-K

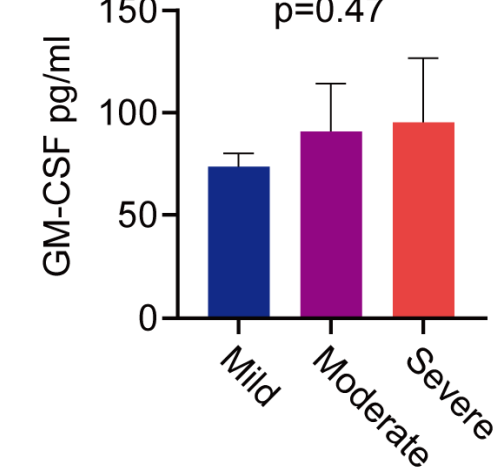

**B-A**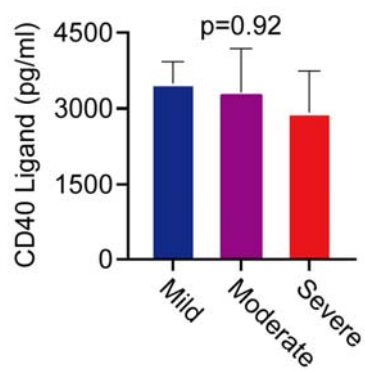**B-B**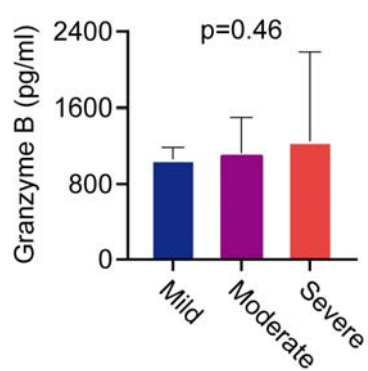**B-C**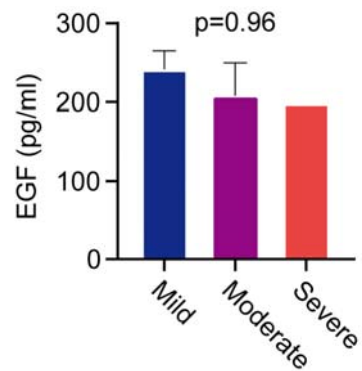**B-D**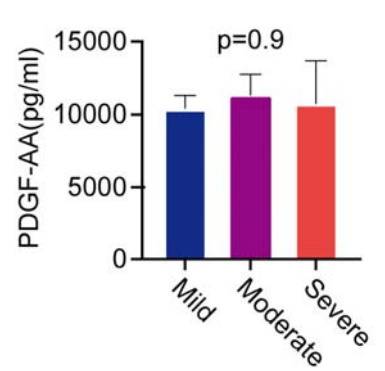**B-E**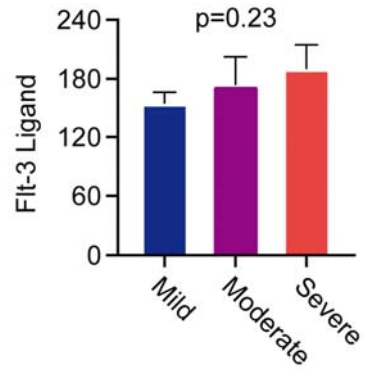**B-F**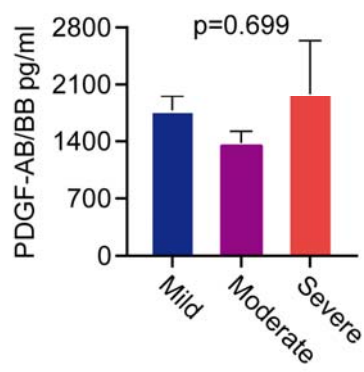

C-A

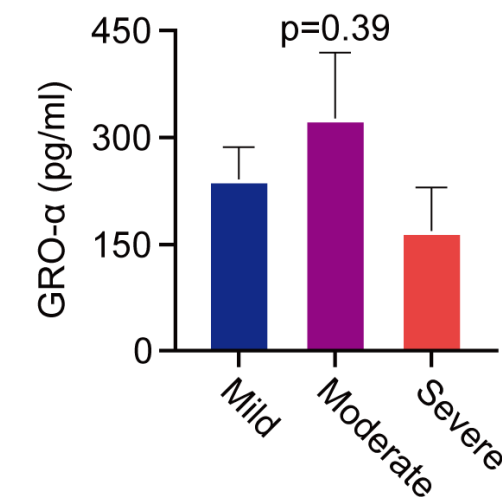

C-B

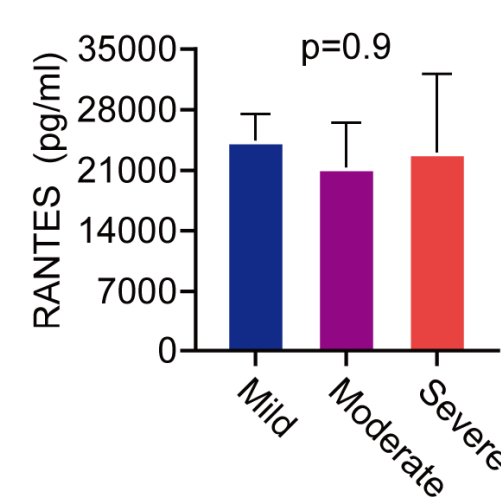

C-C

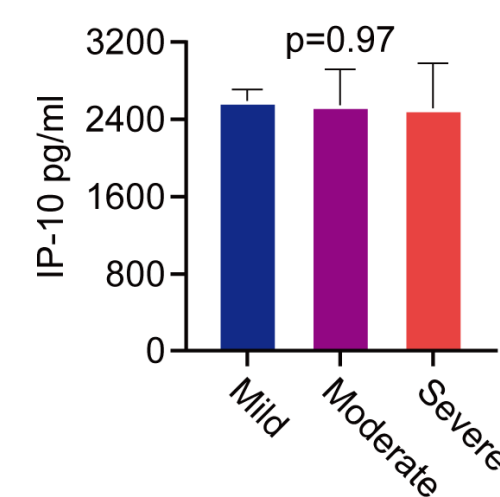

C-D

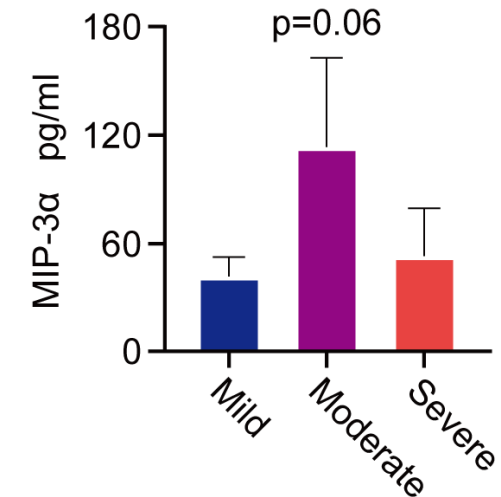

C-E

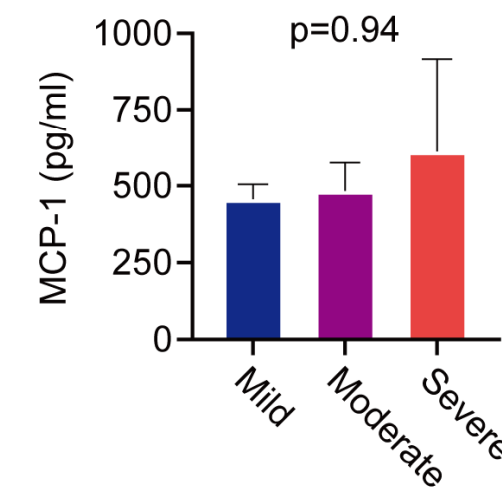

C-F

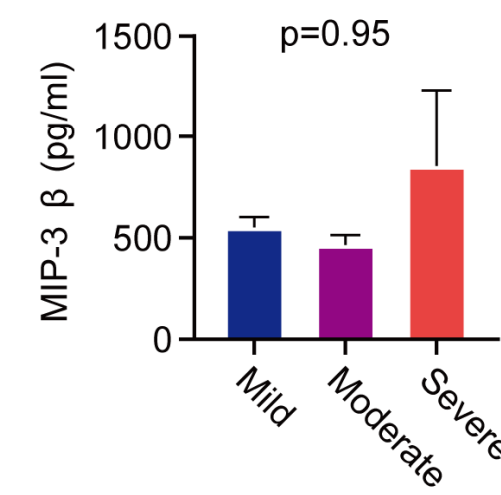

Supplement: Figure S5 — Cytokines lack severity discrimination. [file jcm.01633-25-s0005.pdf]

A-A

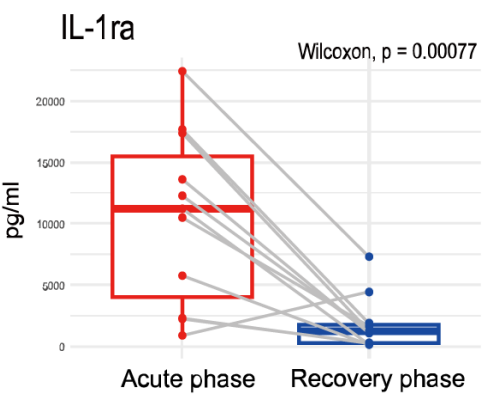

A-B

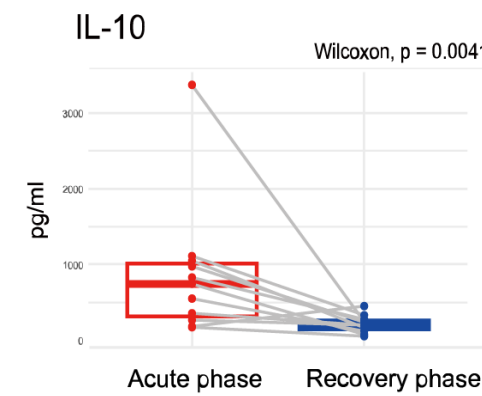

A-C

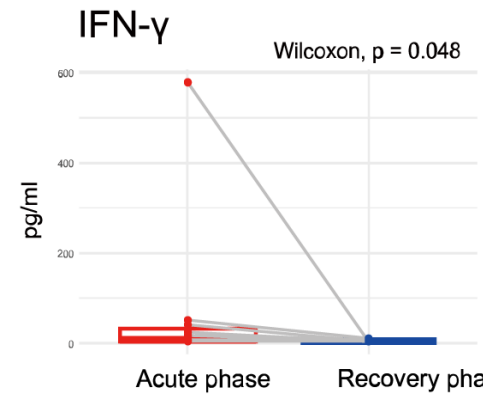

A-D

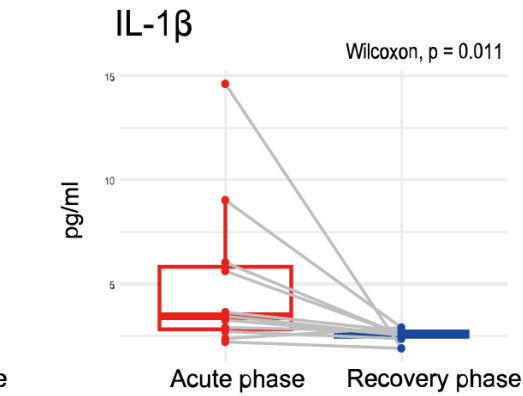

A-E

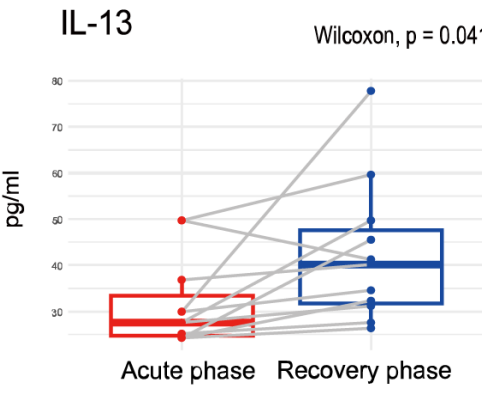

A-F

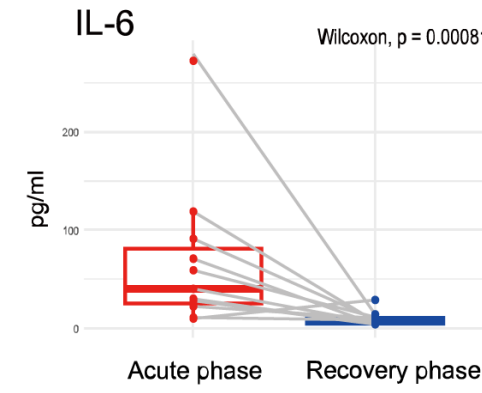

A-G

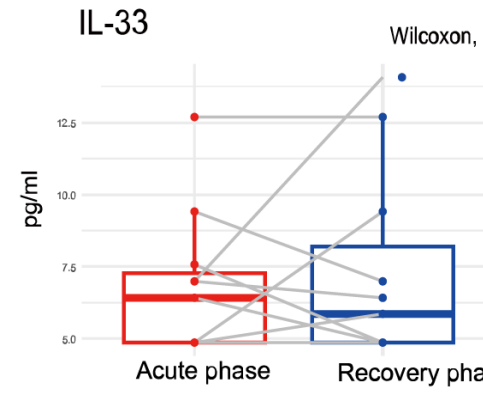

A-H

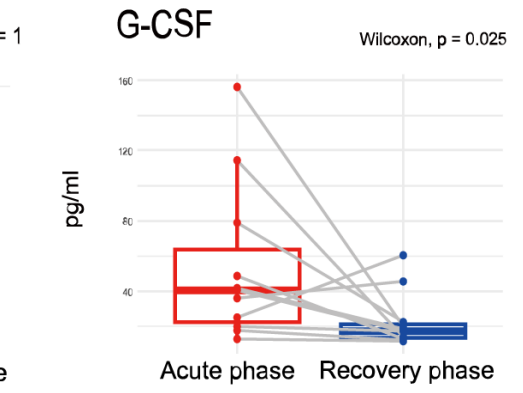

A-I

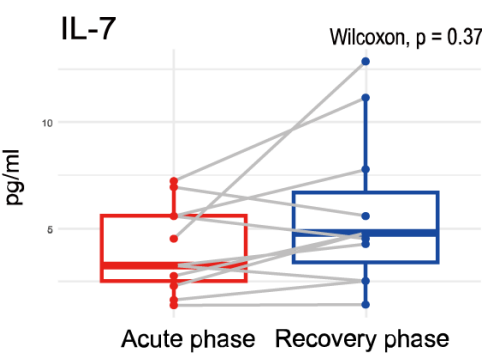

A-J

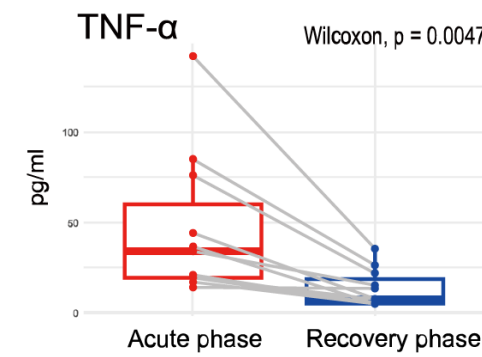

A-K

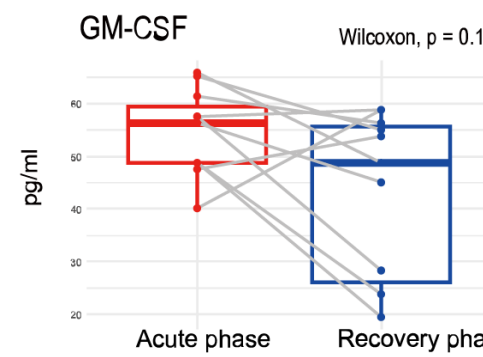

B-A

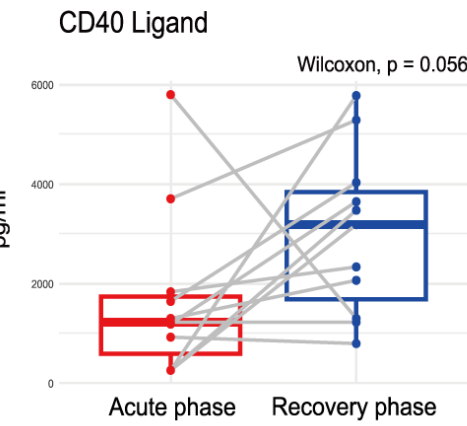

B-B

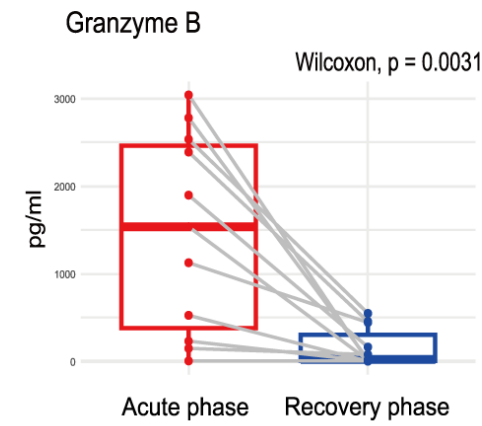

B-C

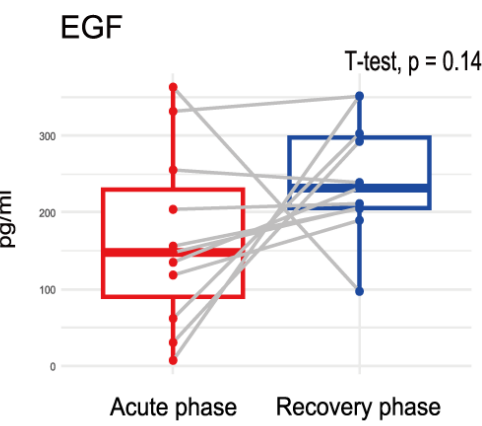

B-D

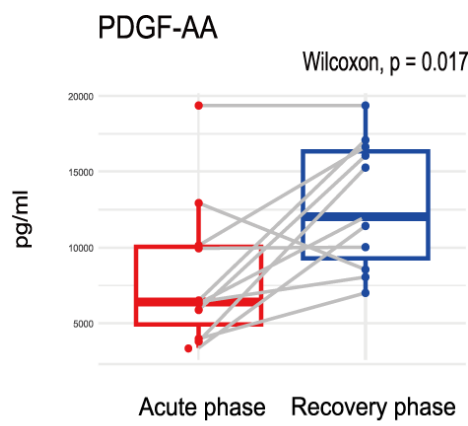

B-E

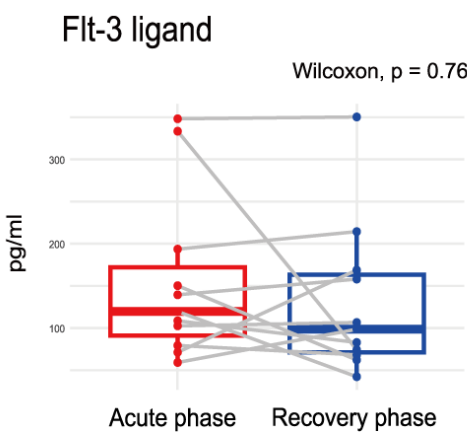

B-F

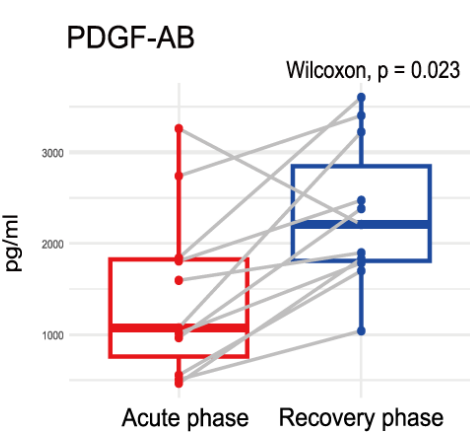

C-A

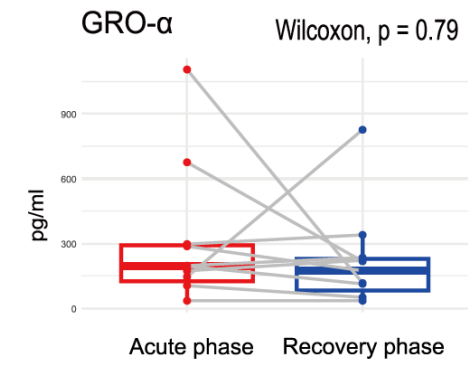

C-B

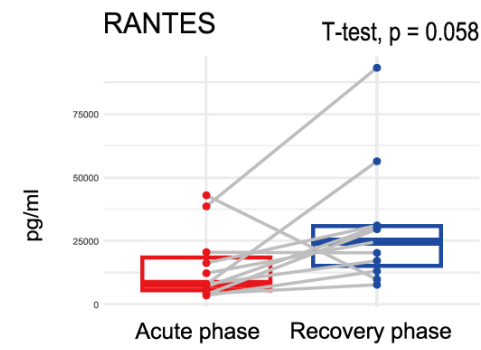

C-C

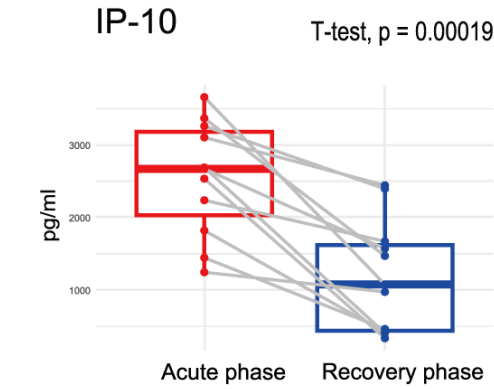

C-D

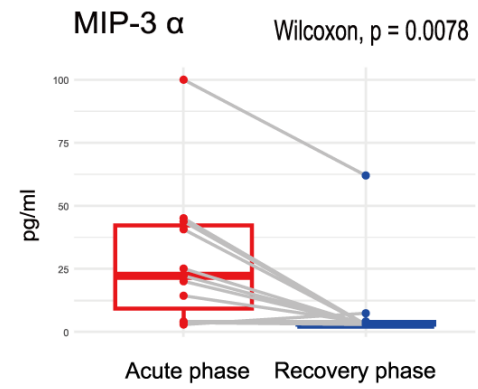

C-E

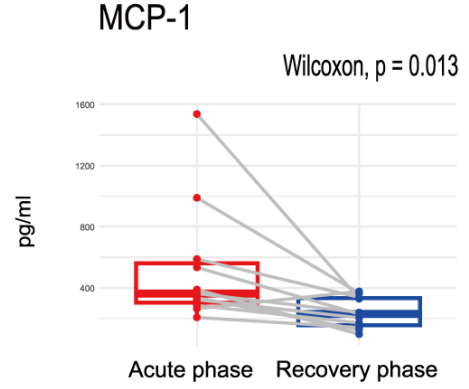

C-F

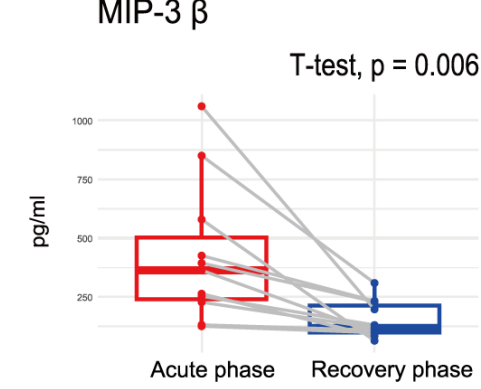

Supplement: Figure S6 — Cytokine phase dynamics. [file jcm.01633-25-s0006.pdf]
