# Supplementary material for: Cytokine and chemokine profiles linked to early severity of scrub typhus: multicenter validation of soluble PD-L1
Source: J Clin Microbiol. 2026 Apr 27;64(6):e01633-25. doi: 10.1128/jcm.01633-25 (PMC13251376; doi:10.1128/jcm.01633-25)
Supplement: Tables S1 and S2 — Table S1: Clinical characteristics of patients with organ dysfunction. Table S2: Hepatic and renal function indices across disease severity groups. [file jcm.01633-25-s0007.docx]

Supplementary table 1. Clinical characteristics of patients with organ dysfunction.

| Systemic organ failure | Renal failure | Hepatic dysfunction | Central Nervous System | Respiratory dysfunction | Circulatory dysfunction |
| --- | --- | --- | --- | --- | --- |
| Number | 4 | 5 | 4 | 3 | 3 |
| Age | 46.5±11.6 | 43.2±12.4 | 44.8±28.8 | 66.7±9.6 | 55.7±23.8 |
| Male sex-No. (%) | 4 (100%) | 4 (80%) | 1 (25%) | 4 (33.3%) | 0 (0%) |

Supplementary table 2. Hepatic and renal function indices across disease severity groups.

|  | Liver | | | | | Kindy | |
| --- | --- | --- | --- | --- | --- | --- | --- |
|  | Dbil | TBil | Ibil | ALT | AST | ALB | Scr |
| Mild | 3.7±2 | 13.4±7.3 | 7.8±4.6 | 90.5±67.4 | 95.7±52.1 | 36.9±4.4 | 92.4±30 |
| Moderate | 35.8±19.7 | 56.3±28.4 | 20.5±9.1 | 217±135.9 | 458.2±655 | 57.1±56.5 | 1276.9±2239 |
| Severe | 47.3±33.4 | 58.7±44 | 11.7±10.5 | 84.5±25 | 140.2±107.6 | 27.7±6.2 | 245.5±160.5 |

Dbil: Direct Bilirubin; TBil: Total Bilirubin; IBil: Indirect Bilirubin; ALT: Alanine Aminotransferase; AST: Aspartate Aminotransferase;

ALB: Albumin; Scr: Serum Creatinine.
