# Supplementary material for: Cytokine and chemokine profiles linked to early severity of scrub typhus: multicenter validation of soluble PD-L1
Source: J Clin Microbiol. 2026 Apr 27;64(6):e01633-25. doi: 10.1128/jcm.01633-25 (PMC13251376; doi:10.1128/jcm.01633-25)
Supplement: Supplemental material legends — Legends for Figures S1 to S6, and Tables S1 and S2. [file jcm.01633-25-s0008.docx]

**Supplementary Figure 1.** Non-Significant Cytokine Comparisons. No significant differences in serum levels of TGF-α, Eotaxin, GRO-β, IL-8, MIP-1β, IL-2, or TRAIL between scrub typhus patients and controls (all p > 0.05; Wilcoxon or paired t-test as appropriate).

**Supplementary Figure 2.** Decreased TRAIL, IL-8, and MIP-1β levels during recovery from scrub typhus. No significant differences in TRAIL, IL-8, or MIP-1β between scrub typhus patients and controls at baseline (Supplementary Figure 1); however, within-patient comparisons revealed significant decreases from acute to recovery phase: TRAIL (*p*=0.001), IL-8 (*p*=0.022), and MIP-1β (*p*=0.001) (Wilcoxon or paired t-test as appropriate).

**Supplementary Figure 3.** No significant changes in TGF-α, Eotaxin, GRO-β, or IL-2 between acute and recovery phases (all *p*>0.05; Wilcoxon or paired t-test as appropriate).

**Supplementary Figure 4.** Association between *Ot* load and inflammatory markers in scrub typhus. All variables showed non-normal distributions (Kolmogorov-Smirnov, all *p*< 0.05). Spearman correlations were performed within severity groups: A. *Ot* bacterial load vs. PCT: Significant positive correlation in all groups (mild: *ρ*=0.48, *p*< 0.001; moderate: *ρ*=0.47, *p*< 0.001; severe: *ρ*=0.62, *p*<0.001); overall intergroup difference: *p*=0.03. B. *Ot* load vs. FR-CRP: No significant correlation in any group (all *p*>0.10); no intergroup difference (*p*=0.12). C. *Ot* bacterial load vs. WBC: No significant correlation in any group (all *p*>0.20); no intergroup difference (*p*=0.71). PCT, but not FR-CRP or WBC, shows consistent association with pathogen burden across disease stages.

**Supplementary Figure 5.** Cytokines Lack Severity Discrimination.

Panel A: All analyzed growth factors showed non-significant differences across disease severity groups (all *p*> 0.05).

Panel B: Chemokines (eg: MCP-1 and RANTES) similarly failed to stratify severity (all *p*>0.05). Panel C: No significant intergroup differences for IL-1ra (*p*=0.12), IL-10 (*p*=0.2), IFN-γ (*p*=0.52), or TNF-α (*p*=0.18) across severity groups.

**Supplementary Figure 6.** Cytokine Phase Dynamics.

Panel A: IL-6 (*p*=0.001), IFN-γ (*p*=0.048), and TNF-α (*p*=0.047) showed significant acute-recovery differences (Wilcoxon).

Panel B/C: Growth factors (eg: PDGF-AB/BB, p=0.02) and chemokines (eg: IP-10, *p*< 0.001) exhibited phase-specific changes (paired t-test/Wilcoxon).

**Supplementary table 1.** Clinical characteristics of patients with organ dysfunction.

**Supplementary table 2**. Hepatic and renal function indices across disease severity groups.
